# South West Orthopaedic Club, November 1990 Meeting

**Published:** 1991-03

**Authors:** 


### South West Orthopaedic Club

### Meeting held at the Postgraduate Centre, Southmead Hospital, Bristol, Saturday, 3rd November, 1990

### CT SCANNING OF THE OS CALCIS: THE IMPLICATIONS FOR INTERNAL FIXATION OF FRACTURES

**D. M. Eastwood, R. M. Atkins**
Dept. of Orthopaedics, University of Bristol

Operative management of displaced intra-articular fractures of the os calcis may be improved by increased understanding of the pathological anatomy. Radiographs and coronal CT scans of 31 fractures were studied.

Radiographs demonstrated 18 joint depression and 13 tongue fractures: oblique views showed calcaneocuboid joint involvement.

CT scans depicted three fragments. The primary fracture line always crossed the subtralar joint. The sustentacular fragment was undisplaced relative to the talus. The lateral fragment was undisplaced relative to the medial articular surface in 11 cases, depressed in 13, and displaced upwards in 7. The mean displacement was 4 mm (range 2–9 mm). In 21 cases the fragment was in valgus (mean 21 degrees, range 5–40).

The lateral wall consisted of the lateral joint fragment in 8 cases, the body fragment in 9 (when the lateral fragment was not in valgus), or by both fragments in 14 cases.

The body fragment was impacted in 27 cases (mean 6 mm, range 3–12 mm) and in varus in 22 cases (mean 14 degrees, range 5–30).

Operative management must reduce the body and lateral joint fragments separately onto the sustentacular fragment. Through a lateral approach: the lateral fragment is identified and rotated outwards. The body fragment is disimpacted and rotated. The medial wall is reduced and held witha temporary coronal K wire. The lateral joint fragment is reduced; held with a lag screw and the 3 major fragments stabilized with a lateral Y-shaped plate.

### THE USE OF RAYON OVERGLOVES TO PREVENT NEEDLESTICK INJURIES

**Dai Jones, S. Paramaswaran**
Morriston Hospital, Swansea

There is general cocnern about the possibility of transmission of disease, particularly HIV and hepatitis, by glove breakage during surgery. Simple double gloving has not been as effective as hoped. In this study rayon dermatological gloves were used over ordinary gloves (Regent) for a period from December 1988 until June 1989 in the practice of one surgeon. A large range of surgical procedures were undertaken. The gloves were tested by inflation with water and the puncture sites were recorded. There was a 22.5 puncture rate in the outer gloves of the double gloves and a 7.5 puncture rate in the inner gloves of the double rubber gloves. This was reduced to 5% on using outer rayon gloves.

Rayon gloves were found to be excellent in preventing tearing as when dealing with wires, but not effective in preventing needle penetration.

### IN VITRO MEASUREMENT OF BONE-CEMENT INTERFACE PRESSURES

**E. J. Smith*, A. J. Ward#, M. Halawa#, A. Aziz#, J. W. Barlow*#**
* Bristol Royal Infirmary, Bristol
### Mount Gould Orthopaedic Hospital, Plymouth
*# Department of Mechanical Engineering, Polytechnic South West, Plymouth

Early aseptic femoral stem loosening remains the commonest mode of failure in cemented total hip replacements. The microinterlock at the bone-cement interface is the key to osseous integration. This is influenced by cementation techniques.

This study experimentally measured bone-cement interface pressures at cement injection and femoral stem insertion, as well as shear strength. We compared three different methods to determine which was the most effective. [1] Early cement injection and late stem insertion. [2] Late cement injection and early stem insertion. (Medium viscosity cement was used with methods [1] and [2]). [3] High viscosity cement.

Twelve cadaver femurs were used in the experiment, using a double tapered collarless prosthesis (Exeter) and a standard bone preparation.

The results showed a statistical difference at the proximal and distal transducers between methods [1] and [2] at cement injection. During stem insertion no statistical difference was recorded, but maximum pressures were lower with method [3]. Bone-cement interface shear strengths were significantly higher in method [1] when compared with the other methods at the level of the proximal and middle transducers.

The quality of the mechanical interlock is based on maintaining adequate pressure from the time of cement injection to stem insertion with a modern standard of bone preparation. This was confirmed in our in vitro study with early cement injection and late stem insertion.

### ELECTRONIC DETECTION OF GLOVE PUNCTURES DURING ORTHOPAEDIC SURGERY

**A. J. Hamer**
Bristol

There is a particularly high incidence of glove punctures in trauma and routine orthopaedic surgery. It is disturbing that in 50% of cases the surgeon may be unaware that a perforation has occurred. A device has been described whereby a surgeon may be warned as soon as he is in direct contact with the patients body fluids, such as occurs if a glove is punctured or if a wet gown touches the patients would (1).

The device has been used prospectively in eighty orthopaedic cases. The alarm sounded 47 times in 27 cases, the maximum number per case being 5. Glove punctures were responsible on 16 occasions, 8 when double gloved, and damp gown sleeves touching the wound caused 30 alarms. The device alarmed only once without explanation.

1. HAMER, A. J. (1987) Electronic device for the detection of breaches in asepsis during surgical procedures. *Br J Surg.* **74**, 1038–1039.

### SAFETY AND RELIABILITY OF THE ABERDEEN SPLINT IN THE NEONATE

**M. Bishay, Dai Jones**
Morriston Hospital, Swansea

Efficacy of screening for congenital hip displacement and hazards of resultant splintage of babies remains controversial.

In this study we are assessing a screening programme for a fixed population in Swansea District over a 2 year period in which ultrasound was the main adjunct to clinical examination.

We particularly looked into the effectiveness of the Aberdeen splint in preventing late case presentation in those babies with questionable congruity.

Avascular necrosis was not a significant complication in this series and there was no psychological or physical complications related to the splint.

### THE FATE OF THE SPASTIC DIPLEGIC HIP

**J. Travlos, E. B. Hoffman**
Capetown

In order to assess the efficiency of adductor and flexor release operations a retrospective study of a long term follow up of flexor and adductor release surgery since 1970 in 40 ambulant spastic diplegic patients was carried out. Only patients older than 8 years and with a longer than 3 year follow up were selected. 27 patients who fulfilled the criteria were also available for follow up. The average age at follow up was 17.7 with a range of 8–26 years and average duration of follow up was 9.2 years. All patients were assessed clinically and radiologically. An AP pelvis and the Reimers migration percentage were used to assess subluxation, and the lateral sacrofemoral angle to assess the compensatory gait pattern. Surgery was performed to improve gait and no hips showed radiological signs of sublaxation.

Adductor release involving adductor tenotomy and gracilis myotomy adequately improved the scissoring gait and only 10% had required repeat operations. Operations involving the adductor brevis muscle, ie, cuting the muscle or an anterior obturator neurectomy leads to an ungainly or hyperabducted gait in over 75% or 6/8 patients.

50% had a residual flexion deformity and the compensatory gait pattern (crouch or lordosis) was confirmed by the radiological sacrofemoral angle. The late flexor release surgery (all after 7 years of age) was thought to be the cause of the residual flexion deformity. Psoas tenotomy only (not involving iliacus led to hip flexion weakness in only 20% of hips.

A significant gait abnormality with retraction of the pelvis and a circumducting gait due to femoral neck ante-version was present in 50% of patients. There was, however, no definite correlation between flexion deformity and femoral neck anteversion.

A significant gait abnormality with retraction of the pelvis and a circumducting gait due to femoral neck ante-version was present in 50% of patients. There was, however, no definite correlation between flexion deformity and femoral neck anteversion.

### METALLIC WEAR DEBRIS IN TOTAL HIP REPLACEMENT

**V. G. Langkamer**
Comparative Orthopaedic Research Unit, Bristol University

Metal corrosion products and wear debris were common findings in metal on metal arthorplasty and were incriminated in loosening. (Charovsy, 1973). The advent of low friction arthroplasty has focussed on the significance of polyethylene debris. The introduction of low modulus metal alloys such as Titanium alloyh (Ti–6Al–4V) have been based on the favourable mechanical properties and biocompatability (Galante, 1983). Discolouration of soft tissues adjacent to the implant "metallosis" have been attributed to the corrosion of the impant and of no serious consequence. In-vivo studies have suggested that titanium is stored in the reticuloendothelial system for twelve months but aluminium corrosion products do not plateau even after nine years. The clinical significance of this is unknown. (Woodman, 1985). The situation when corrosion is accompanied by mechanical wear is less well known.

We present a patient in whom mechanical wear of a titanium alloy prosthesis has been demonstrated by open synovial biopsy and present histological, metallurgical and immunological data. An in-vivo specimen with a long term prosthesis made of a similar alloy has been shown to have wear debris in the macrophages of the local tissues as well as the deep lymph nodes implying a wide dissemination. Titanium has been associated with platelet deficiency (Carroll, 1968). Aluminium has been known to inhibit bone formation and reach toxic levels in renal impairment. Prolonged immobilisation of these patients has been associated with dementia (in Woodman, 1983).

The meeting consisted of clinical presentations in the morning and a guest lecture by Professor Allen Goodship, entitled "Osteoathritis in other animals".

The South West Orthopaedic Club prize was presented to Mr A J Hamer for his paper entitled "Electronic detection of glove punctures during orthopaedic surgery". The presentation was made by Mr Harry Griffiths. The X-ray quiz was won by Mr Graham Taylor, Lecturer in Orthopaedic Surgery University of Bristol.

The club was greatly saddened at the death of one of its senior members Mr John Baily, and a short period of silence was observed in his memory.

